# Decannulation protocol in pediatric patients: case series study

**DOI:** 10.1590/1984-0462/2025/43/2023187

**Published:** 2024-09-23

**Authors:** Luciana Diniz Gomide de Miranda, Luiza Araujo Alves Borges, Laura Caldeira Zavaglia, Tereza Cristina Lara Mesquita, Luanna Rodrigues Leite, Larissa Tavares Aguiar, Isabela Furtado de Mendonça Picinin

**Affiliations:** a Fundação Hospitalar do Estado de Minas Gerais, Hospital Infantil João Paulo II, Serviço de Assistência Integral à Criança Traqueostomizada, Belo Horizonte, MG, Brazil.; b Faculdade de Ciências Médicas de Minas Gerais, Belo Horizonte, MG, Brazil.

**Keywords:** Tracheostomy, Pediatrics, Nasal obstruction, Weaning, Traqueostomia, Pediatria, Obstrução nasal, Desmame

## Abstract

**Objective:**

The aim of this study was to describe the phases of a decannulation protocol and the results from its application in hospitalized children.

**Methods:**

This is a retrospective, observational study. Data were collected from medical records of decannulated patients followed up in a pediatric hospital in Belo Horizonte, Minas Gerais between 2011 and 2021.

**Results:**

Among the children followed up in the service (n=526), 23% (n=120) were successfully decannulated. Children aged between 2 months and 16 years, with a mean age of 4 years, 69% of whom were male, were evaluated. About 75% of the patients have tracheostomy due to upper airway obstruction and 60% of these due to acquired subglottic stenosis. At the beginning of the decannulation protocol, 5.5% of the patients had moderate oropharyngeal dysphagia, while 80.4% had normal swallowing. Correction in the upper airway pre-decannulation was performed in 39.5% of the patients, dilation in 63.8%, and endoscopic correction was performed in 55.3%. After performing the decannulation, none of the patients had complications.

**Conclusions:**

The described decannulation protocol is safe, since no complications such as death and need for recannulation happened.

## RESUMO

**Objetivo:**

Descrever as etapas de um protocolo de decanulação e os resultados da aplicação desse protocolo em crianças hospitalizadas.

**Métodos:**

Trata-se de um estudo observacional retrospectivo. Os dados foram coletados de prontuários de pacientes decanulados acompanhados em um hospital pediátrico de Belo Horizonte, Minas Gerais, entre 2011 e 2021.

**Resultados:**

Foram analisadas 120 crianças com idade entre dois meses e 16 anos, com média de idade de quatro anos, 69% das quais eram do sexo masculino. Cerca de 75% dos pacientes apresentam traqueostomia por obstrução de vias aéreas superiores e 60% destes por estenose subglótica adquirida. No início do protocolo de decanulação, 5,5% dos pacientes apresentavam disfagia orofaríngea moderada, enquanto 80,4% apresentavam deglutição normal. A correção da pré-decanulação das vias aéreas superiores foi realizada em 39,5% dos pacientes, a dilatação em 63,8% e a correção endoscópica em 55,3%. Após a realização da decanulação, nenhum paciente apresentou complicações.

**Conclusões:**

O protocolo de decanulação descrito é seguro, pois a taxa de complicações como óbito e a necessidade de recanulação foi ausente.

## INTRODUCTION

Tracheostomy is currently indicated in several areas of medicine, including pediatrics. It is performed, for example, in cases of upper airway anomalies, both congenital and secondary to prolonged intubation, and in children who need long-term mechanical ventilation due to respiratory failure.^1^ Despite being an important and often necessary intervention strategy, there exist some risks and complications associated with tracheostomy.^1^ Tracheostomy carries a high risk of mortality and associated complications such as hemorrhage, stoma infection, pneumothorax, and subcutaneous emphysema.^2^ Therefore, timely removal of the tracheostomy tube is important.

The process of removing the tracheostomy tube to restore spontaneous breathing is called decannulation.^3^ It is recommended to start as early as possible to promote better patient recovery and reduce the risk of complications.^4^ However, premature or improper decannulation, without adequate monitoring, can lead to potential consequences, such as respiratory failure, loss of airway patency, and even death.^5^ Therefore, a thorough evaluation of the patient is necessary to guide decision-making and ensure a safe and effective decannulation.

Despite the associated risks, decannulation in children lacks standardization of assessment, and there is no universally accepted protocol. Consequently, this procedure varies significantly among different institutions.^5^ Currently, in Brazil, there is no established guideline outlining the steps for determining the indication for decannulation and achieving optimal outcomes. Therefore, it is crucial to develop a clear protocol with defined steps to ensure an adequate and secure airflow for patients. Thus, the objective of this study, a single-center case series, is to describe the results of implementing a decannulation protocol in hospitalized children.^6,7^

## METHOD

This is a retrospective, descriptive, observational study. The study adhered to the STROBE guidelines.^7^ The present study was approved by the Research Ethics Committee 5.337.226. Data were collected from the medical records of patients who underwent decannulation and were followed up at a public hospital between 2011 and 2021.

The inclusion criteria were tracheostomized patients who were followed up by the Integral Assistance Service for Tracheostomized Children (SAIT) and who demonstrated good cough effectiveness. Good cough effectiveness was defined as the individual’s ability to protect the airways by eliminating secretions and foreign bodies from the lung.^8,9^ The effectiveness of the cough was assessed by the physiotherapist using an expiratory flow meter (Peak Flow) to measure the peak cough flow (PFT) in collaborative patients. For successful decannulation, the PFT needed to be greater than 160 L/min, preferably close to 270 L/min.^10^ Another inclusion criterion was the number of aspirations, which could not exceed two in the last 8 h.^11^ This criterion was evaluated by the physiotherapist.

The exclusion criteria were patients with unfavorable endoscopic evaluation of the airways, such as subglottic stenosis with obstructions above 60–70% (Stenose Grading Scale grade II–III of Cotton-Cincinnati Children´s Pediatric Otolaryngology) or glottic stenosis, as well as other airway obstructions. Additionally, patients were excluded if they had a severe swallowing disorder (saliva aspiration), neurological diseases, dependence on mechanical ventilation for more than three months, or dependence on tracheostomy for pulmonary hygiene.

The protocol described in this article considered the same factors that contraindicate decannulation at the First Clinical Consensus and National Recommendations on Tracheostomized Children of the Brazilian Academy of Pediatric Otorhinolaryngology (ABOPe) and Brazilian Society of Pediatrics (SBP), which include absence of endoscopic airway evaluation, dependence on mechanical ventilation in the last 3 months, and dependence on tracheostomy for pulmonary toilet.^12^

An evaluation form was developed for data collection by a trained examiner, including sex, date of birth, date of tracheostomy, date of admission to the service, date of initiation of the decannulation protocol, date of approval for decannulation, indication of tracheostomy, presence of comorbidities, type of cannula used at admission and at the time of decannulation, presence of dysphagia, changes in bronchoscopy, functional assessment with an occluded cannula, submission to a progressive adaptation of the speaking valve, submission to a progressive occlusion protocol, nocturnal saturation measurements, polysomnography, fiberoptic nasolaryngoscopy, pre-decannulation airway correction, decannulation site, post-decannulation complications, presence of persistent tracheocutaneous fistula, and decannulation date.

**Figure 1:**
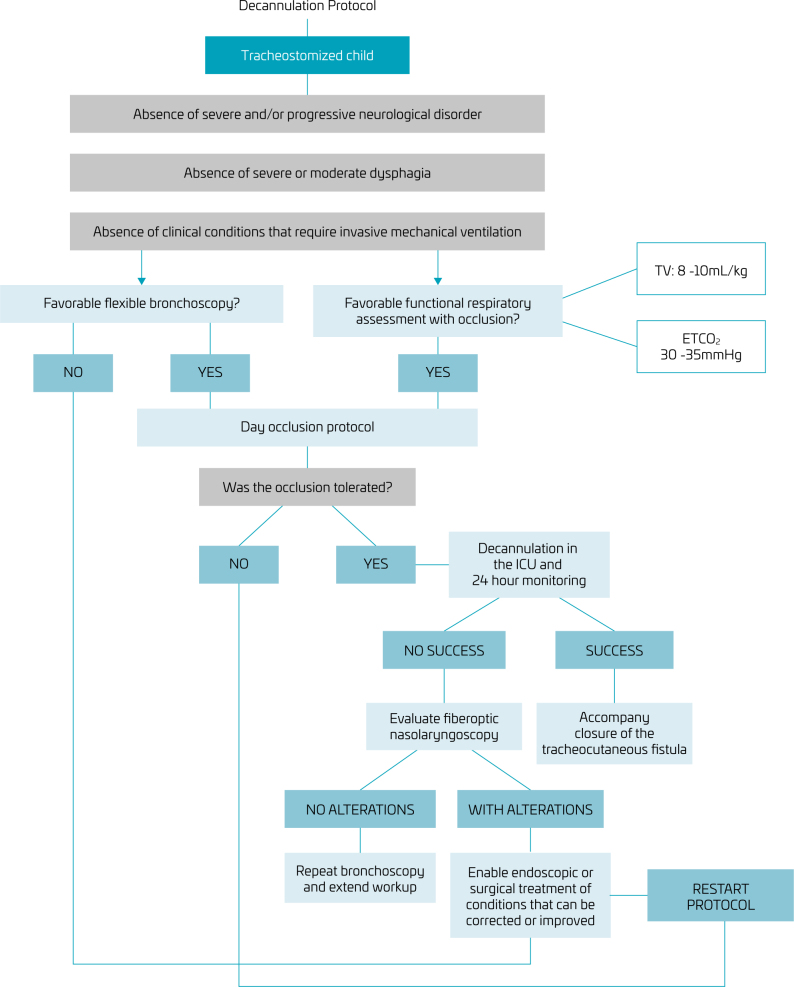
Decannulation protocol criteria.

TV: Tidal volume; ETCO_2_: exhaled CO_2_; ICU: intensive care unit; PSG: polysomnography.

The decannulation protocol is presented in Figure 1. The protocol included several components, such as bronchoscopy, capnography, ventilometry, progressive occlusion of the tracheostomy tube, and assessment of dysphagia. The application of this protocol involved a multidisciplinary team consisting of a pediatric pulmonologist, a bronchoscopist, physiotherapists, speech therapists, psychologists, nurses, and nursing technicians. The team includes an experienced pediatric pulmonologist who conducts thorough clinical investigations to identify any lung abnormalities that could impede or hinder the decannulation process. Imaging tests, such as chest X-rays or computerized tomography scans, are used when necessary. The role of this team is crucial in identifying factors that may compromise the success of decannulation, such as ineffective cough, with difficulty in eliminating secretions, swallowing disorders with salivary aspiration, hypertrophy and collapse of soft tissues, exacerbated during sleep, and hypotonia of the thoracic muscles leading to hypoventilation.

To initiate the protocol, the medical team is responsible for a detailed clinical evaluation to exclude pulmonary and neurological diseases, such as severe cerebral palsy. Subsequently, laryngoscopy and bronchoscopy are performed to assess the presence of granulation tissue, suprastomal collapse, or any other problem that may interfere with decannulation.

The speech therapy team is responsible for the clinical evaluation of swallowing in the tracheostomized patients, identifying changes in the dynamics of swallowing, considering the stages of development of the stomatognathic system, and performing the blue dye test, which consists of a procedure for coloring saliva/food with blue food dye, in order to identify the aspiration of saliva/food in tracheostomized individuals. It is important for the decannulation protocol to rule out moderate to severe dysphagia.^13^ Although the blue dye test is not the best approach, it can provide important additional information in cases where, even after a thorough clinical evaluation, there is concern about tracheal aspiration of saliva and/or food. In addition, the team follows the American Speech-Language-Hearing Association (ASHA) recommendations for patients who fall into the risk group for the test. These are patients with increased gastrointestinal permeability, including sepsis or septic shock; burns; trauma; kidney failure; esophageal and/or gastrointestinal surgical interventions; celiac disease and inflammatory bowel disease; and allergies to dyes. In addition, the team of pediatric pulmonologists assessed clinical manifestations of salivary and/or food aspiration by monitoring pulmonary symptoms, such as bronchial hyperreactivity, increased secretion volume, and coughing during or after feeding.

Finally, the physiotherapist provides objective parameters that assist in the decannulation process. Physiotherapists perform ventilatory functional tests using capnography, ventilometry, and progressive occlusion of the tracheostomy tube. Capnography is performed through the patient’s mouth with the tracheostomy tube occluded.^14^ Ventilometry is performed using the Wright Mark 8 Analog Ventilometer device. Tidal volume is measured using the equipment connected to the tracheostomy tube and through the mouth with the cannula occluded. An acceptable tidal volume is greater than 5 mL/kg, preferably ranging from 8 to 10 mL/kg.^15^ Physiotherapists assess the patient’s respiratory function by evaluating the degree of hypoxemia and hypercapnia. The monitored parameters include peripheral oxygen saturation levels above 90–94%, depending on the underlying disease, an arterial partial pressure of oxygen (PaO_2_) above 60 mmHg, and an arterial partial pressure of carbon dioxide (PaCO_2_) between 35 and 45 mmHg. It is also assessed whether the patient tolerates well occluding the tracheostomy tube, without a significant drop in baseline oxygen saturation or an increase in baseline respiratory or heart rates. Progressive occlusion allows the patient to experience a “less artificial voice.” In some cases, it may be necessary to reduce the size of the cannula, except in infants, where the presence of the cannula, even if it is small, can still cause significant airway obstruction.^16^

Some factors are necessary for the patient to be considered eligible for decannulation. These factors include the following: favorable bronchoscopy, favorable lung functional measures, tolerance to occlusion of the tracheostomy tube, and absence of moderate to severe dysphagia. After conducting all these assessments, the patient undergoes a sleep assessment using overnight oximetry with the tracheostomy tube occluded. This assessment aims to gather additional data on tolerance to nocturnal occlusion in children with suspected respiratory issues during sleep, such as snoring and night sweats. Overnight, specific data, including oxygen saturation, heart rate, and respiratory rate, as well as the patient’s protective patterns, are monitored. In the morning, the results are reviewed to identify any significant changes. Whenever central or obstructive sleep apnea is suspected, a polysomnography examination is requested, since sleep apnea may impact the success of decannulation, particularly in children with upper airway hypotonia (e.g., children with Down’s syndrome). This additional test is not essential but provides valuable information regarding readiness for decannulation. However, it is expensive and may not always be available in the public health system. Its use is also not universally agreed upon among the existing protocols. Decannulation can be attempted on the tracheostomized patient under intensive care supervision. In children with suspected sleep-related problems, polysomnography for a more comprehensive evaluation is considered.^13,14,15^ However, this test is not routinely conducted in our service due to its limited availability in the public health system and the small number of referral centers able to perform it on children. As a result, intra-hospital oximetry with an occluded tracheostomy tube has been used for sleep analysis in children undergoing the decannulation process. Consequently, polysomnography is reserved for selected cases in which nocturnal oximetry alone is deemed insufficient/inconclusive.

In this study, the descriptive measures minimum, maximum, median (Q2), quartiles (Q1 and Q3), mean, standard deviation (SD), and confidence interval of the mean were presented to describe quantitative variables, while absolute (n) and relative (%) frequencies were used for categorical variables.

## RESULTS

In the present study, 120 out of 526 children followed up in this service were decannulated and included in this study. They were children and adolescents between 2 months and 16 years of age with a mean age of 4 years, 69% of whom were male. The most frequent indication for tracheostomy was upper airway obstruction, 60% of which was due to acquired subglottic stenosis. At the beginning of the decannulation protocol, 5.5% of the patients had moderate oropharyngeal dysphagia, while 80.4% had normal swallowing. At the time of decannulation, there was no severe neurological impairment in any of the patients, and none of them had significant dysphagia resulting from an uncontrolled neurological condition. Table 1 presents the clinical characteristics of the children.

**Table 1:** General characteristics of the study patients.

Variables	Data
Age (in years), *mean±standard deviation [minimum–maximum]*	3.9±4.0 [0.2–16.1]
Gender, male, n (%)	69.0 (57.5)
Indication for tracheostomy, n (%)	
Prolonged mechanical ventilation	31.0 (26.7)
Upper airway obstruction	75.0 (64.6)
Acquired subglottic stenosis	60.0 (51.7)
Laryngomalacia	6.0 (5.2)
Congenital subglottic stenosis	3.0 (2.5)
Tracheomalacia	1.0 (0.9)
Papillomatosis	5.0 (4.3)
Neurological disorder	7.0 (6.1)
Craniofacial anomaly	0.0 (0.0)
Others	3.0 (2.6)
Presence of dysphagia-Beginning of the decannulation protocol, n (%)	
Normal swallowing	74.0 (80.4)
Mild oropharyngeal dysphagia	13.0 (14.1)
Moderate to severe oropharyngeal dysphagia	5.0 (5.5)
Time from tracheostomy to SAIT (in months), *mean±standard deviation [minimum–maximum]*	11.3±18.2 [0.0–95.0]
Time between admission to SAIT and decannulation (in months), *mean±standard deviation [minimum–maximum]*	21.2±23.2 [0.0–127.0]

SAIT: Integral Assistance Service for Tracheostomized Children.

All patients underwent bronchoscopy at least once, and 59% more than once, due to the alterations found. It is important to emphasize that, at the time of decannulation, any detected anatomical alterations had been resolved or mitigated.

Table 2 presents the data regarding bronchoscopic and lung functional assessments with the occluded cannula. Among the patients studied, 55% of them underwent functional assessment, with an average tidal volume (TV) of 11 mL/kg. The mean ETCO_2_ was 27 mmHg. At the time of the functional assessment, the mean oxygen saturation was 97%. It was observed that 10% of the patients used a speaking valve. Regarding the sleep study, monitoring nocturnal oxygen saturation was necessary in 42% of the patients, while polysomnography was necessary in only 5% of the patients.

**Table 2:** Description of patients regarding bronchoscopic and functional assessments with the cannula occluded.

Variables	Result
Functional evaluation with occluded cannula?	
*Yes*, n (%)	55 (45.8)
- If yes, tidal volume, mL/kg, *median (Q*_1_ *–Q*_3_ *)*	11.1 (9.1–14.7)
- If yes, ETCO_2_, mmHg, *median (Q*_1_ *–Q*_3_ *)*	27.0 (23.0–28.0)
- If yes, SpO_2_ in room air, %, *median (Q*_1_ *–Q*_3_ *)*	97.0 (96.0–98.0)
Subjected to progressive speech valve adaptation?	
- *Yes*, n (%)	11 (9.3)
Submitted to nocturnal oximetry?	
- *Yes*, n (%)	50 (42.0)

In 39.5% of the patients, procedures to correct the airway were needed before decannulation, and 55.3% of these were endoscopic, of which 63.8% involved dilation. Patients undergoing endoscopic dilation underwent the procedure on average four times. In almost all cases, 98.3%, decannulation took place in an intensive care setting. It is important to note that decannulation was successful in all patients, with no need to reestablish the tracheostomy in any patient who was eligible for the decannulation protocol.

Pre-decannulation cannula caliber reduction was performed in 21.9% of patients. The mean time between performing the tracheostomy and admission to the SAIT was 4 months, and the mean time between admission to the SAIT and decannulation was 14 months.

## DISCUSSION

The present study describes the decannulation protocol used in tracheostomized children in a Brazilian single center. Among the children followed up in the service (n=526), 23% were successfully decannulated. It’s important to note that the decannulation rate in the service is limited because the hospital is a referral center for rare diseases. As a result, a significant number of tracheostomized patients have severe neurological impairments, requiring a tracheostomy for airway protection, rendering them ineligible for decannulation. Pozzi et al., in their decannulation protocol, also demonstrated no decannulation failure.^4^ However, unlike ours, their protocol included a prolonged period of hospitalization lasting up to months before decannulation, which may further assure the underlying conditions of the airways.^4^ As mentioned before, decannulation in children is not standardized, there is no universally accepted protocol, and this procedure varies considerably between institutions.^5^ Pediatric tracheostomy decannulation studies usually include less than 80 patients, which highlights the relevance of our study and can guide other services succeed when decannulating children with tracheostomy.^15^

Decannulation success rates ranged from 67 to 94% in previous reports. Mitchell et al.^17^ have proposed pre-decannulation recommendations for children, which include criteria similar to those used in our study. These criteria involve the absence of ventilatory support for a minimum of 3 months prior to decannulation, the absence of aspiration events that could mandate a tracheostomy for adequate pulmonary hygiene, and documentation of a patent airway through flexible laryngoscopy. Failure rates for decannulation vary from 6.5 to 21.4%, as described in a review on the topic. Mahadevan et al.^18^ studied 122 tracheotomized patients younger than 16 years. Decannulation was carried out successfully in 92 patients (75%), although 6 (6.5%) subsequently required recanalization. The study by Leung et al.,^19^ with patients aged less than 20 years old, highlights how difficult decannulation is. In total, 12 patients died, and 30 of the 53 survivors were decannulated (median cannulation time: 123.5 days), with a decannulation rate success of only 46%.

Some patients underwent cannula caliber reduction to optimize peri-cannula airflow and favor breathing through the anatomical airway. After a satisfactory functional assessment, a progressive occlusion protocol is initiated to help condition the child to breathe through the nose and mouth. In this process, it may be necessary to use the speaking valve, as an intermediate resource, important for respiratory conditioning. With this device, air is inhaled through the tracheostomy but exhaled through the upper airways. All these steps are carefully discussed as a team, taking into account the family’s expectations, focusing on the child’s comfort, which is essential to the success of decannulation.

According to the literature^17,20,21^ and our results, most children were tracheostomized due to upper airway obstruction, mostly subglottic stenosis. Performing endoscopic dilation in tracheostomized patients reestablishes airflow through the anatomical airway, so that the patient is decannulated in a less invasive and faster way.^22,23^ In this way, surgical correction is avoided, which is a more complex procedure, usually performed in multiple stages, and it tends to postpone the decannulation.^24,25^

When sleep-related problems are suspected, polysomnography is relevant.^26,27,28^ Polysomnography is not a resource used in our decannulation protocol, as it is a high-cost tool and is often not available in public health services. The non-use of polysomnography in our routine is in line with that of Seligman et al.,^23^ as these authors explain that polysomnography is a limited resource and has a high cost that can reach US$600 to US$1,700. Furthermore, they add that although some consensus states that the apnea/hypopnea index and desaturation events evaluated in the polysomnography exam are predictors for successful decannulation, it should not be disregarded that nocturnal oximetry is also an exam. which accurately detects desaturation events, and indirectly, apnea/hypopnea episodes. Seligman et al.,^23^ Hang et al.,^27^ and Tsai et al.^28^ consider that nocturnal oximetry is a viable alternative to polysomnography, as it also detects obstructive events during the sleep of a child in the process of decannulation, which is in line with what we use in our protocol.

Performing a polysomnography with an occluded cannula, although recommended by some services, was not recommended by the First Clinical Consensus and National Recommendations on Tracheostomized Children of the Brazilian Academy of Pediatric First Clinical Consensus and National Recommendations on Tracheostomized Children of the Brazilian Academy of Pediatric Otorhinolaryngology (ABOPe) and Brazilian Society of Pediatrics (SBP).^12^ Wirtz et al.^29^ mention that the use of this routine is still considered uncertain. There are doubts about what we can consider a favorable polysomnography to indicate decannulation, as patients with mild and even moderate obstructive apnea can have a successful decannulation, as long as it is well monitored by a specialized multidisciplinary team. Gurbani et al.^30^, cited by Wirtz,^29^ carried out a study comparing the use of bronchoscopy with polysomnography to indicate decannulation and found that 26% of participants who had polysomnography considered “not favorable” were able to be successfully decannulated. The authors add that polysomnography is not a routinely used tool, that is, it is a valid test, but it provides more accurate information in more selectable and complex cases.

Despite the effectiveness of the proposed protocol, it is important to discuss alternative approaches in the event of potential failures in the process, particularly considering the potential expansion of patients and adoption by other services. If the attempt at decannulation is unsuccessful, despite a favorable bronchoscopy and functional evaluation, it is considered appropriate to expand the diagnostic assessment to evaluate other factors that may affect proper airflow through the upper airways.

It is suggested to consider performing fiberoptic nasolaryngoscopy (to better evaluate the upper airway with the patient awake), polysomnography (for a more comprehensive assessment of sleep-related apnea, whether obstructive or central), as well as nocturnal oximetry. If the initial workup does not reveal any abnormalities, it is necessary to repeat the bronchoscopy to conduct a fresh anatomical assessment of the airways and review any obstructive factors, particularly those that may arise from prolonged use of the tracheostomy, such as granuloma and tracheal stenosis. If the additional diagnostic tests indicate abnormalities, it is important to consider endoscopic or surgical interventions to correct or improve the conditions.^30^ This should be done through collaboration and discussion with other medical teams.

As a limitation of this study, some variables were included over the years, after having already started collecting information from patients, given the evolving scientific knowledge on the decannulation process. Hence, some data were missing at the beginning of the study period. Another limitation of the present study is the absence of an otorhinolaryngologist as part of the decannulation team. The fluoroscopic video of swallowing (VFSS) and the fiberoptic endoscopic evaluation of swallowing (FEES) are not available in the public health system. Furthermore, the study only included data from a single hospital, which restricts the generalization of the results. Future studies should include diverse samples from multiple healthcare facilities.

In conclusion, the protocol described herein was both safe and effective for decannulation of hospitalized children. Future studies should investigate the applicability of this protocol to other populations.

## Data Availability

The database that originated the article is available with the corresponding author.
